# Innovative CRISPR Screening Promotes Drug Target Identification

**DOI:** 10.1021/acscentsci.2c01142

**Published:** 2022-10-27

**Authors:** Xinyu Ling, Tao Liu

**Affiliations:** † Chemical Biology Center, Department of Molecular and Cellular Pharmacology, Pharmaceutical Sciences, State Key Laboratory of Natural and Biomimetic Drugs, Peking University, 38 Xueyuan Road, Haidian District, Beijing 100191, China

Target identification of small molecules to decipher their mechanism of action is an important process in drug development. However, the unraveling of cellular drug targets is often complicated, time-consuming, and labor intensive. To address these issues, several methods have been developed.^1,2^ For example, one approach uses affinity-based biochemical screening with chemical probes, usually a structural derivative of the drug, to pull down the target protein from the cell lysate, which is then identified by proteomic analysis. However, the small molecule must be chemically modified with a tag that does not significantly influence its activity, and the proteomic identification of the target protein is difficult. Functional genomic screening tools, on the other hand, provide an unbiased solution without the need to synthetically prepare active chemical probes. Among these tools, CRISPR-based screening techniques have proven to be the most powerful, specific, and efficient under most circumstances. Typically, CRISPR screening is based on a pooled CRISPR guide RNA library, which is introduced into the target cell by lentiviral transduction, making it possible to efficiently identify target genes based on gRNA sequencing. Such pooled CRISPR screening largely relies on positive selection after offsetting the antiproliferative effects of the drugs and thus cannot be applied to nonantiproliferative small molecules. In a recent issue of *ACS Central Science*, Jingxin Wang and co-workers^3^ established a loss-of-function CRISPR screening platform for target identification of a nonantiproliferative drug candidate by linking the compound activation pathway with the expression of a suicide gene. Using this platform, they pinpointed STING as the target of BDW568, a small molecule IFN-I activator, and identified a key metabolizing enzyme, CES1, that activates BDW568 in cells.

**Figure 1:**
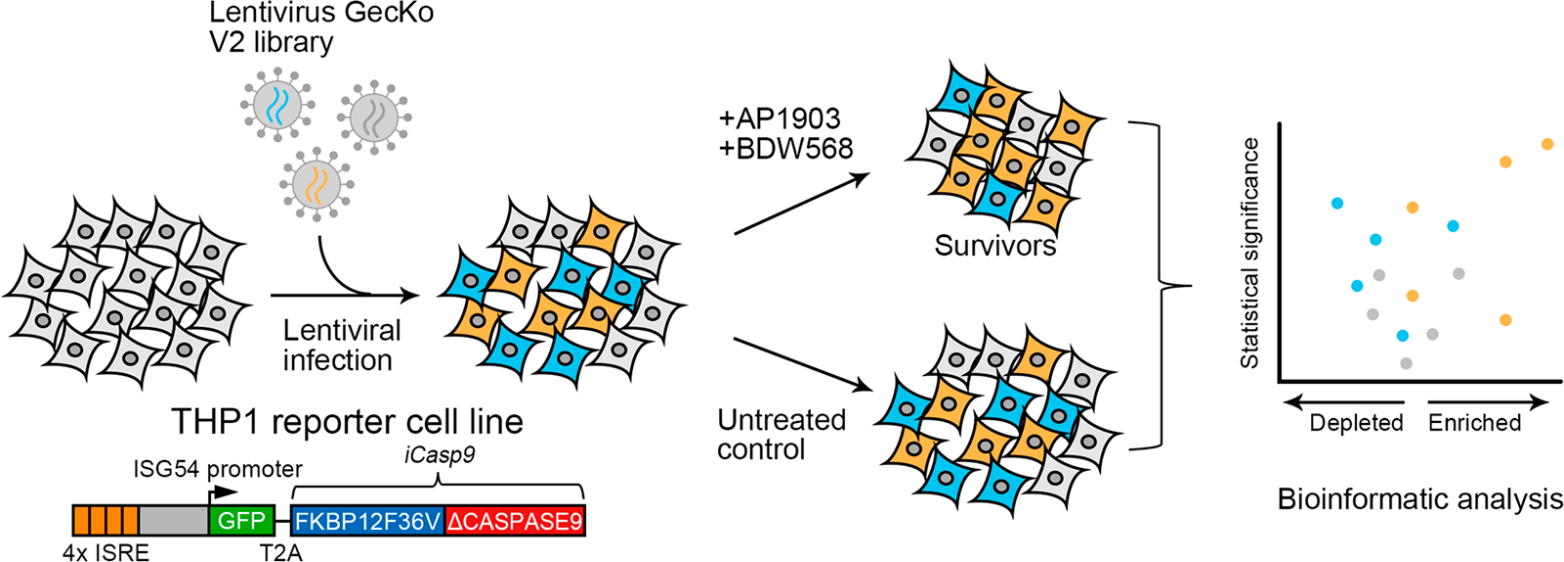
CRISPR screening workflow for target identification of IFN-I activator BDW568.

CRISPR-based drug target identification works by generating a selectable phenotype upon compound treatment, usually based on proliferation assays. BDW568 is a potent IFN-I signaling activator; however, its mechanism of action is not based on proliferation. Therefore, a traditional CRISPR-based selection system cannot be used to identify drug targets of BDW568. To overcome this limitation, Wang and co-workers designed a general selection system by coupling compound signaling with inducible suicide gene expression. Since BDW568 is an IFN-I activator, suicide gene iCasp9 was placed under the control of interferon-sensitive response element (IRSE); thus, the cytotoxic activity was triggered upon BDW568 treatment. To make the screening more stringent and eliminate false positives, the expression of iCasp9 was also under the control of a small molecule, AP1903, so that the expression time could be further fine-tuned. (Figure 1) The authors used a lentiviral GeCKO gRNA library targeting 19,050 genes to conduct such loss-of-function CRISPR screening. Among the top hits, three genes including STING, CES1, and SEC24C were further confirmed to show a correlation with the small molecule activation. Further analysis demonstrated that BDW568 is indeed dependent on and specific to STING.

Interestingly, the authors observed a significant enrichment of CES1 in the screening and confirmed that the hydrolysis of BDW568 carboxyl ester by CES1 is an important step for the compound activation to achieve stimulation of the STING pathway. The cGAS-STING signaling pathway is a key component of the innate immune system. Agonists for STING have shown promise in treating cancer and other diseases. In addition, this finding further demonstrates the specificity and efficiency of CRISPR screening in drug target identification, revealing its capacity to identify not only the activation pathway-related proteins but also key metabolic enzymes involved in the drug’s metabolism.

Transcription activator or suppressor small molecule identification is one of the major techniques used for drug development. Although this work used cell live-death as a model to prove the general utility of the platform, future iterations can explore more diverse phenotypic screening signals. The key to successful CRISPR-based screening is to design an assay to enrich or isolate cells based on the phenotypic response upon gene knockout or activation. While traditional phenotypic screening is based on proliferation, some other phenotypes such as expression of a fluorescent reporter gene for FACS-based selection, expression of a marker protein for affinity-based isolation, and cell migration or differentiation-based enrichment can also be used as a selection readout.^4^ In the past few years, CRISPR has revolutionized the genetic screening of small molecule targets, strongly promoting drug discovery. With the fast development of CRISPR methods, more powerful tools have been established^5^ such as CRISPR interference (CRISPRi), CRISPR activation (CRISPRa), and base editor and RNA editing. These state-of-art technologies have a strong potential to be applied as high-throughput and sensitive methods to probe the mechanism of action of small molecules and transform drug discovery.

Wang’s work provides an elegant selection platform for target identification by linking transcription activation to a suicide gene, which could greatly extend the screening scope of CRISPR, making it adaptable to a broad range of drugs with nonproliferative activities.
